# Recent Progress on Two-Dimensional Carbon Materials for Emerging Post-Lithium (Na^+^, K^+^, Zn^2+^) Hybrid Supercapacitors

**DOI:** 10.3390/polym13132137

**Published:** 2021-06-29

**Authors:** Chao Han, Xinyi Wang, Jian Peng, Qingbing Xia, Shulei Chou, Gang Cheng, Zhenguo Huang, Weijie Li

**Affiliations:** 1 Institute for Superconducting and Electronic Materials, AIIM Building, Innovation Campus, University of Wollongong, Wollongong, NSW 2522, Australia; ch861@uowmail.edu.au (C.H.); xw734@uowmail.edu.au (X.W.); jp647@uowmail.edu.au (J.P.); qx366@uowmail.edu.au (Q.X.); shulei@uow.edu.au (S.C.); 2 School of Civil and Environmental Engineering, University of Technology Sydney, Sydney, NSW 2007, Australia; 3 School of Chemistry and Environmental Engineering, Wuhan Institute of Technology, Donghu New & High Technology Development Zone, Wuhan 430205, China; gchenglab@163.com

**Keywords:** 2D carbon materials, sodium-ion hybrid capacitor, potassium-ion hybrid capacitor, zinc-ion hybrid capacitor, challenges and prospects

## Abstract

The hybrid ion capacitor (HIC) is a hybrid electrochemical energy storage device that combines the intercalation mechanism of a lithium-ion battery anode with the double-layer mechanism of the cathode. Thus, an HIC combines the high energy density of batteries and the high power density of supercapacitors, thus bridging the gap between batteries and supercapacitors. Two-dimensional (2D) carbon materials (graphite, graphene, carbon nanosheets) are promising candidates for hybrid capacitors owing to their unique physical and chemical properties, including their enormous specific surface areas, abundance of active sites (surface and functional groups), and large interlayer spacing. So far, there has been no review focusing on the 2D carbon-based materials for the emerging post-lithium hybrid capacitors. This concept review considers the role of 2D carbon in hybrid capacitors and the recent progress in the application of 2D carbon materials for post-Li (Na^+^, K^+^, Zn^2+^) hybrid capacitors. Moreover, their challenges and trends in their future development are discussed.

## 1. Introduction

**Figure 1:**
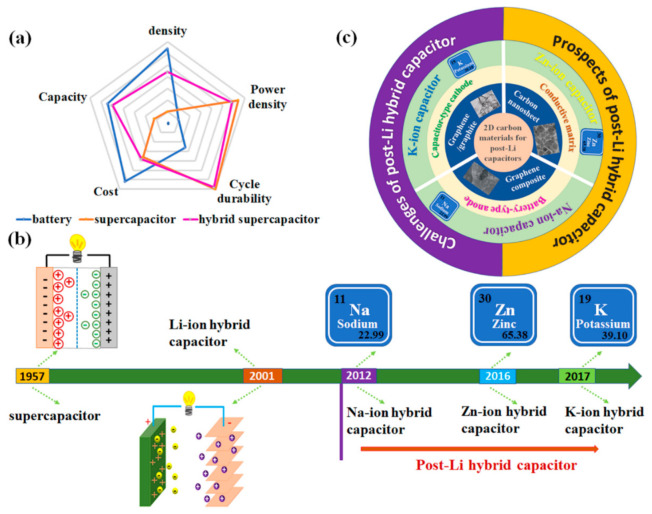
(**a**) Comparison of the features of the battery, supercapacitor, and hybrid capacitor. (**b**) Historical timeline for capacitors. (**c**) The theme of this review.

The depletion of fossil fuel and global warming are forcing human to reduce the utilization of fossil fuel and cut carbon dioxide (CO_2_) emissions. Many countries in the European Union announced a prohibition on the sale of oil-fuelled automobiles, which will commence from 2025 and be completed in 2040. This has been impelling the rapid development of electric vehicles, which must be accompanied by the development of energy storage devices. Such energy storage devices must possess both high energy density (related to the mileage) and high specific power density (related to charging time and acceleration time). The state-of-the-art lithium-ion batteries (LIBs) can deliver a high specific energy density of 200 Wh kg^−1^, but their specific power density is relatively low (<350 W kg^−1^). In contrast, the commercial supercapacitors show high power density of up to 10 kW kg^−1^ and long-term durability through the reversible adsorption/desorption of ions on the electrode surface but have low specific energy densities (<5 Wh kg^−1^). To obtain both high energy density and power density, the lithium hybrid ion capacitor (HIC) emerged in 2001 to bridge the gap between LIBs and supercapacitors [1]. The HIC is a hybrid electrochemical energy storage device that combines the intercalation mechanism of a lithium-ion battery anode with the double-layer mechanism of the cathode. Thus, HICs combine the high energy density of batteries and the high power density of supercapacitors (Figure 1a) [2].

Due to the increase in the cost of lithium precursors and unevenly distributed lithium resources, some alternative batteries for LIBs have been developed recently [2], such as sodium-ion storage batteries (Na-O_2_, Na-ion, Na-S, etc.) [3,4,5,6,7,8,9,10,11], potassium-ion batteries (K-O_2_, K-ion battery, etc.) [12,13,14,15,16], and zinc-ion batteries (Zn-air, aqueous zinc-ion batteries, etc.) [17,18,19,20,21]. Soon afterwards, the corresponding hybrid capacitors were reported. The sodium-ion hybrid capacitor (NIC) has attracted tremendous research interest since 2012, when Chen et al. reported sodium-ion pseudocapacitors based on a hierarchically porous nanowire composite [22]. The first hybrid potassium-ion capacitor (KIC) prototype was proposed by Azais’s group in 2017, which contained a positive active carbon electrode and a graphite negative electrode (Figure 1b) [23]. Unlike the above-mentioned hybrid capacitors, the zinc-ion hybrid capacitor (ZIC) consists of a zinc anode, with its operation based on Zn^2+^ deposition/stripping instead of the intercalated/de-intercalated anode [24,25,26].

Carbon materials are widely utilized in the field of energy storage due to their low cost, light weight, and easy recovery. Especially in capacitors, carbon materials function as the various vital constituent parts, such as the activated carbon/porous carbon/graphene for capacitive-type cathodes [27,28,29,30,31,32], graphite/graphene/disordered carbon/N-doped carbon nanotubes for battery-type anodes [33,34,35,36,37,38], or graphite oxide as a filler for the gel electrolyte [39]. The capacitor cathode requires the carbon materials to have ample active sites for reversible anion adsorption/desorption. For the battery-type anode, expanded interlayer spacing is needed for reversible insertion/extraction of large Na^+^, K^+^, or Zn^2+^ ions. Moreover, it was reported that oxygen-containing functional groups on the carbon not only provide extra capacitance, but also expand the interlayer spacing of the carbon and thus improve the diffusion of K^+^ [28].

**Figure 2:**
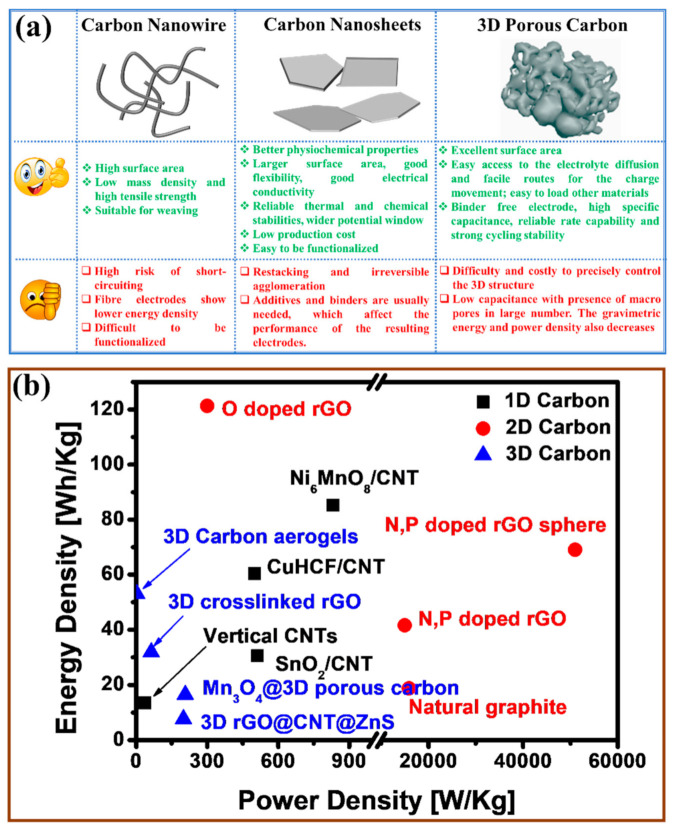
(**a**) Comparison of 1D/2D/3D carbonaceous materials when used in supercapacitors. (**b**) Ragone plot of some typical 1D [40,41,42,43], 2D [33,44,45,46], and 3D [47,48,49,50] carbon-based supercapacitors; rGO is reduced graphene; CNT is carbon nanotubes; CuHCF is copper hexacyanoferrate.

Figure 2a compares the advantage and disadvantage of the carbonaceous materials with different morphologies, from which it is easy to know that compared with 1D fiber structure and 3D porous structure, 2D morphology is much more suitable for energy storage systems due to its better physicochemical properties, good flexibility, good electrical conductivity, reliable thermal and chemical stabilities, wider potential window, low production cost and availability of abundant surface functional groups [51,52]. Moreover, the 2D structure exhibits higher energy density and power density than 1D and 3D structures (Figure 2b).

To date, there has been no review focusing on 2D carbon-based materials for the emerging post-lithium hybrid capacitors. Therefore, in this conceptual article, we focus on the latest applications of 2D carbon-based materials in emerging post-lithium hybrid capacitors, and the theme of this review is summarized in Figure 1c. First, we describe the energy storage mechanism of hybrid capacitors; then, we summarize the recent progress in the utilization of 2D carbon materials in the field of sodium-ion hybrid capacitors, potassium-ion hybrid capacitors, and zin-ion hybrid capacitors; finally, the challenges and future prospects of 2D carbon materials for post-lithium hybrid capacitors are proposed.

## 2. Basic Knowledge of Hybrid Capacitors

### 2.1. Energy Storage Mechanism of Hybrid Capacitors

**Figure 3:**
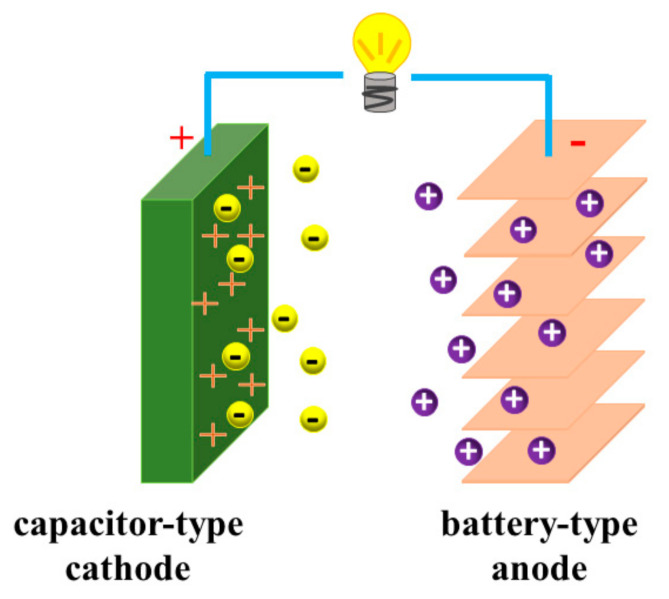
Schematic illustration of the configuration of hybrid capacitors.

Based on the energy storage mechanism, supercapacitors can be classified into three types [2]: (1) electrochemical double-layer capacitors (EDLCs), which store energy through the adsorption of ions on the surfaces of the electrodes; (2) pseudocapacitors (PCs), which have a storage mechanism based on fast surface redox reactions; and (3) hybrid capacitors, which store energy through a combination of the adsorption of ions on the surface of the electrodes and redox reactions of their electrodes. In terms of cell configuration, the capacitors are categorized into two types [2,53,54,55,56,57]: one type includes the symmetric capacitors (such as EDLCs and PCs), in which the same material is used for both electrodes; the other type includes the asymmetric supercapacitors such as hybrid capacitors, as shown in Figure 3. Since the topic of this review is the post-Li hybrid capacitors, we will focus on the energy storage of hybrid capacitors in the following part.

Generally, hybrid capacitors contain both non-faradaic electrodes (based on ion adsorption on their surfaces) and faradaic electrodes (based on redox reactions) [51]. The non-faradaic electrodes act as cathodes, and the faradaic electrodes serve as anodes. During the charging process, the anions from the electrolyte are adsorbed on the surface of the cathode, while the cations take part in the redox reactions on the anode. Conversely, during the discharging process, the anions desorb from the surface of the cathode, and the cations de-intercalate/de-alloy from the anode. Thus, both anions and cations of electrolyte salts take part in the energy storage mechanism of hybrid capacitors, where the anions are involved in the non-faradaic process and the cations take part in the faradaic process. Carbonaceous materials are usually chosen as capacitor-type cathodes, such as active carbon [35,45,46,58,59,60,61,62,63], graphene [64,65], etc., which store energy by adsorption of anions from the electrolyte. The battery-type anode in hybrid capacitors, featuring redox reactions with cations, can be classified into mainly four types, according to their reaction mechanisms, which are related to the particular cations. (i) Insertion type: Generally, the charge carriers insert into the layered structures like lithium-ion batteries, such as graphite [45,66], graphene (or reduced graphene oxide (rGO)) [33,44,46,64,65], Ti oxides (TiO_2_ [67,68,69,70], Li_4_Ti_5_O_12_ [1,71,72], Li_2_Ti_3_O_7_ [73], NaTi_2_(PO_4_)_3_ [65,74]) and spinel-LiMn_2_O_4_ [75,76,77]. (ii) Conversion type: The cations directly react with anode materials. This group mainly contains iron oxides (Fe_3_O_4_ [78], Fe_2_O_3_ [79]). (iii) Alloying type: Including silicon/copper [80], boron-doped Si/SiO_2_/C [81] and Sn@N-rich CNT [82], which store energy by forming alloys with cations. (iv) Plating/stripping type: Such as zinc foil in zinc-ion hybrid capacitors [26,83,84] and Mg foil in Mg-ion hybrid capacitors [85,86].

### 2.2. The Classification of Hybrid Capacitors

Various hybrid capacitors have so far been reported. According to the valence of the cation charge carriers, hybrid capacitors can be divided into two types: one contains alkali-metal-ion (A^+^) hybrid capacitors, including Li-ion hybrid capacitors [46,87,88,89,90,91,92,93,94,95], Na-ion hybrid capacitors [33,34,37,44,45,62,64,66,96,97,98,99,100,101,102,103,104,105] and K-ion hybrid capacitors [35,36,38,58,59,106,107,108,109,110]; the other contains multivalent metal-ion hybrid capacitors, including Zn-ion hybrid capacitors [25,26,27,29,30,31,83,84,111,112,113,114], Mg-ion hybrid capacitors [85,86], Ca-ion hybrid capacitors [115], and Al-ion hybrid capacitors [116,117,118]. These hybrid capacitors can share the same cathode materials, based on the adsorption/desorption of anions onto/from the surface. However, there is a huge difference in the selection of anodes, since the cations take part in the faradaic process on the anodes as stated in Section 2.1. In the case of alkali-metal-ion (Li^+^, Na^+^, K^+^) hybrid capacitors, generally, the anode candidates are carbon materials, Ti oxides, and iron oxides, for which the reaction mechanisms related to the cations are based on intercalation, conversion, and alloying. Metals based on the plating/stripping, however, are not suitable for alkali-metal-ion hybrid capacitors. In terms of multivalent metal-ion hybrid capacitors, except for Al-ion hybrid capacitors, the metals (such as Mg, Zn, and Ca) can be directly used as anodes based on the plating/stripping storage mechanism. For Al-ion hybrid capacitors, the anode materials are usually intercalation-type materials, such as MXene [117], W_18_O_49_ [118], and MoO_3_ [116].

### 2.3. The Roles of 2D Carbon Materials in Hybrid Capacitors

In hybrid capacitors, 2D carbon materials can play several roles, such as cathodes, anodes, and the conductive matrix, which can greatly enhance the kinetics of electrodes. To achieve excellent performance, 2D carbon materials must meet certain requirements. When 2D carbon materials serve as capacitor cathodes, they must have ample active sites for reversible anion adsorption/desorption. To increase the density of active sites, graphene must be prevented from restacking by using some spatial pillars (such as carbon nanotubes (CNTs) or polymer fibres) [35,84,101]. One example is reported by Zhao et al.; by manipulating the interfacial chemistry and interactions between the polyimide and graphene, the sodium-ion storage capacity of the composite was significantly improved, from ∼50 mAh g^−1^ for pure polyimide to 225 mAh g^−1^ for a polyimide−graphene composite [35]. In addition, it has been recognized that foreign atom doping can possibly adjust the electronic structure and induce more edge sites and defects, which is favourable for enhancing the capacity via increasing the adsorption sites for anions [36,46,59,66,82,105,119,120,121]. Wen et al. co-doped graphene with S and N atoms; the supercapacitor based on the doped graphene exhibited superior potassium storage capability with a high capacity of 449 mAh g^−1^ at 0.05 A g^−1^. On the contrary, single S- and single N-doped graphene show lower capacitances of 310 and 183 mAh g^−1^ at the same rate [59].

In the case of battery-type anodes, 2D carbon materials are required to have large enough interlayer spacing for reversible insertion/extraction of large-size Na^+^, K^+^ or Zn^2+^ ions. It has been demonstrated that the interlayer spacing of carbon can be expanded through rich P and N heteroatom doping into the carbon lattice [36]. Also, the heteroatom-doped carbon could also increase the density of adsorption sites for cations [36]. Moreover, functionalized carbon materials could improve capacitor performance by expanding the interlayer spacing and enhancing adsorption sites for cations. For example, oxygen-functionalized carbon nanofibers could store more K ions through $-C=O+e^{-}+K^{+}↔-C-O-K$, and the incorporation of the oxygen-containing functional groups could expand the interlayer spacing of the carbons and thus result in improved K^+^ diffusion [28].

In contrast to the capacitive-type cathodes, battery-type anodes suffer from sluggish cation diffusion. Therefore, 2D carbon materials are generally utilized as conductive matrices to form graphene composite electrodes, enhancing the diffusion kinetics to address the kinetic imbalance between the two electrodes [60,61,62,63,65,87,108]. For example, Huang et al. reported a hybrid sodium supercapacitor based on an interlayer-expanded MoS_2_/rGO composite. The graphene skeleton frame delivered sufficient charges and the highly interlayer-expanded MoS_2_ achieved fast ion diffusion; the as-prepared composite exhibited excellent performance as the anode material for sodium capacitor, delivering 580 mAh g^−1^ capacitance at 100 mAh g^−1^ [87].

**Figure 4:**
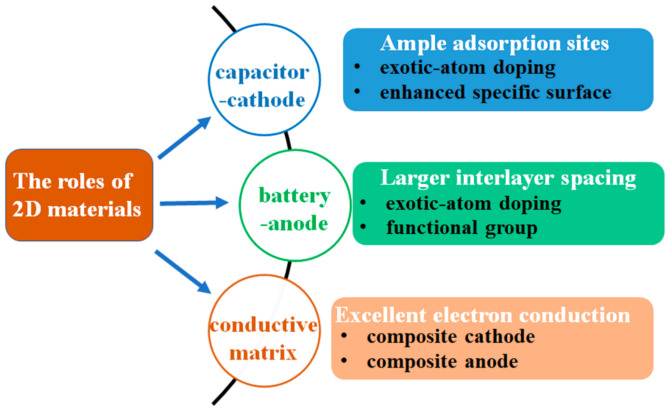
The roles of 2D carbon materials in hybrid capacitors.

The roles of 2D carbon materials in hybrid capacitors and their relevant requirements are summarized in Figure 4. In the following parts, we will discuss the application of 2D carbon materials in the post-Li hybrid capacitors in detail.

## 3. Graphite/Graphene for Post-Li Hybrid Capacitors

### 3.1. Graphite/Graphene as Anode for Post-Li Hybrid Capacitors

**Figure 5:**
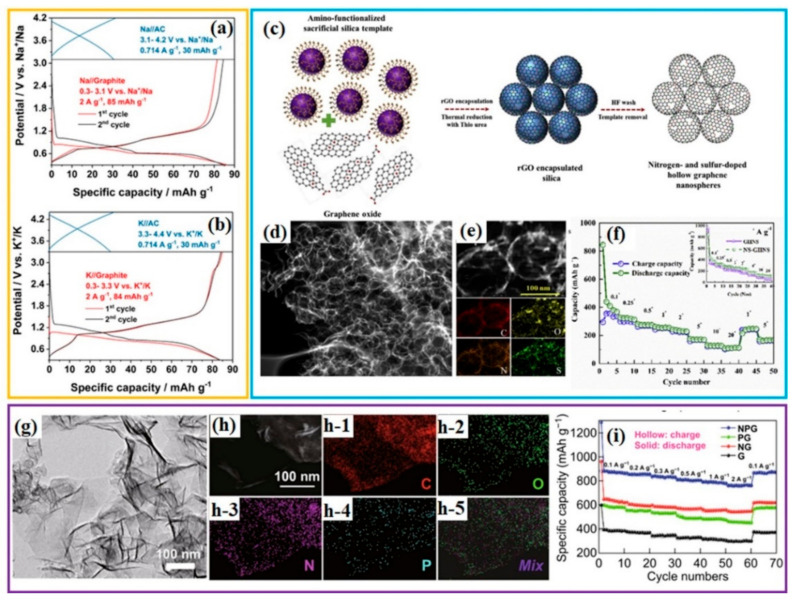
Graphite/graphene as anode for post-Li (Na^+^, K^+^) hybrid capacitors: charge–discharge curves of graphite as anode in three-electrode (**a**) sodium half-cells and (**b**) potassium half-cells. Reproduced with permission [45]; Copyright 2019, American Chemical Society. (**c**–**f**) hollow sphere composite of N,S-doped graphene (NS-GHNS) for sodium-ion hybrid capacitors: (**c**) schematic illustration of the synthesis of NS-GHNS, (**d**) transmission electron microscope (TEM) image and (**e**) energy dispersive spectroscopy (EDS) mapping of NS-GHNS, (**f**) rate performance of NS-GHNS as anode in sodium half-cells; reproduced with permission [33], Copyright 2020, Elsevier. (**g**–**i**) N,P-doped graphene (NPG) as anode for potassium hybrid capacitors: (**g**) TEM image, (**h**) EDS mapping (h-1 to h-5 represent the distribution of C, O, N, P and all elements), and (**i**) rate performance of NPG at different current densities; (G, NG, and PN stand for graphene, N-doped graphene, and P-doped graphene, respectively) Reproduced with permission [46], Copyright 2019, Springer.

Graphite is a commercial, layered anode material for Li-ion batteries, due to its natural abundance. The application of graphite in anodes for the storage of sodium ions and potassium ions, however, is inhibited by its sluggish kinetics, caused by the strong interactions between graphite and Na^+^/K^+^. Recently, it has been recognized that the co-intercalation of glyme-solvated Na^+^ or K^+^ can enable fast and highly reversible intercalation/extraction of Na^+^/K^+^ into/from graphite [122,123,124]. Inspired by the successful utilization of natural graphite as anode electrode material for sodium-ion batteries and potassium-ion batteries, Liu X. et al. used natural graphite as anode electrode for sodium-ion hybrid capacitors (NICs) and potassium-ion capacitors (KICs). The electrochemical performance of natural graphite as anodes for NIC or KIC was first evaluated in three-electrode cells using the electrolyte of 1 M NaPF_6_ or KPF_6_ in diglyme. The results showed a high initial Coulombic efficiency (ICE) of over 94%, suggesting highly reversible intercalation–deintercalation of diglyme-solvated Na^+^ or K^+^ (Figure 5a,b). Coupled with activated carbon (AC) as cathode, graphite/AC NIC and KIC full cells showed high energy density and power density, with 21.8 Wh kg^−^^1^/17,127 W kg^−1^ and 18.8 Wh kg^−1^/15,887 W kg^−1^, respectively [45].

Graphene is widely utilized as anode electrode for sodium-ion batteries and potassium-ion batteries [125,126,127,128] due to its excellent conductivity and larger interlayer spacing compared to natural graphite. When graphene is used as a battery-type anode in hybrid capacitors, its large interlayer spacing rather than a big surface area is crucial for the capacitive performance. Table 1 summarizes graphene/graphite as anode in post-Li hybrid capacitors. It was recognized that the heteroatoms doped into carbon can both enlarge the interlayer spacing of the carbon and increase the adsorption sites for cations [33,36,46]. Y. S. Lee’s group prepared S, N-doped graphene hollow spheres through the hard template method. Graphene oxide was wrapped around amino-functionalized silica spheres and then thermally reduced with thiourea in a furnace at 700 °C, as illustrated in Figure 5c. After removing the SiO_2_ template, graphene with a hollow sphere morphology was obtained (Figure 5d), which is beneficial for electron and mass transport. Transmission electron microscopy—energy dispersive spectroscopy (TEM–EDS) elemental mapping clearly demonstrated the uniform doping of nitrogen and sulphur on the carbon lattices (Figure 5e). Heteroatom doping can improve the electrical conductivity, resulting in graphene that favours fast surface-mediated Na-ion storage (Figure 5f). In the potassium-ion hybrid capacitor, it was also demonstrated that N,P-doped graphene offered superior rate performance and the largest capacity in comparison with bare graphene and single-atom (N or P)-doped graphene, as shown in Figure 5g–i [46]. However, graphite/graphene as the battery-type anode for hybrid capacitors suffers from kinetic imbalance between the anode and the capacitor-type cathode, so it is necessary to improve its rate performance through heteroatom doping.

### 3.2. Graphite/Graphene as Cathode for Post-Li Hybrid Capacitors

**Figure 6:**
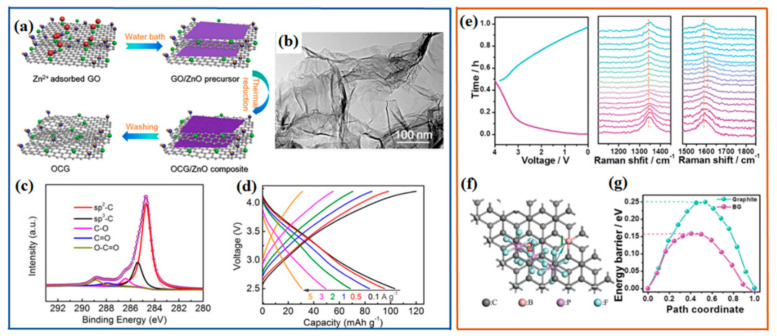
Graphite/graphene as cathode for post-Li capacitors. (**a**–**d**) Oxygen-functionalized graphene (OCG) as cathode for Na-ion hybrid capacitors: (**a**) schematic illustration of the synthesis of OCG, (**b**) TEM image, (**c**) C 1s XPS analysis, and (**d**) charge–discharge curves at different current densities. Reproduced with permission [44], Copyright 2018, Elsevier. (**e**–**g**) B-doped graphite (BG) as cathode for sodium-ion hybrid capacitors: (**e**) in situ electrochemical–Raman spectroscopy test of the BG electrode in an Na-ion hybrid capacitor, (**f**) optimized PF_6_ diffusion in B-doped graphite layers, and (**g**) the corresponding energy barriers from DFT calculations. Reproduced with permission [66], Copyright 2018, Royal Society of Chemistry.

Due to its large specific surface area, graphene is a suitable capacitor-type cathode for hybrid capacitors [65]. Generally, graphene suffers from layer restacking during the reductive synthesis, decreasing the energy density of capacitors. Apart from the specific surface area, oxygen-containing functional groups, especially carbonyl and carboxyl, can contribute to pseudocapacitance through surface redox reactions with cations, which is able to increase the total capacitance of graphene. To obtain oxygen-group functionalized graphene without restacking, X. Zhang et al. used in situ grown ZnO nanosheets as a blocking agent to prevent the graphene layer from restacking during the heat treatment, and then oxygen-rich graphene (OCG) was prepared after removing ZnO (Figure 6a,b) [44]. The high-resolution C 1s XPS spectrum suggested that some oxygen groups were detected on the surface of graphene (Figure 6c), including C–O (286.4 eV), C=O (287.9 eV) and O–C=O (288.9 eV). As a capacitor-type positive electrode, the OCG electrode was evaluated in Na half-cells with a voltage window of 2.5–4.2 V at different current densities (Figure 6d). The charge–discharge curves presented a quasi-linear relationship between the voltage and the capacity, demonstrating dominant electronic double-layer capacitance and some pseudocapacitance contribution. Although the oxygen groups could provide some pseudocapacitance, it should be noted that a much higher content of oxygen functional groups would result in a drop in the conductivity, so that the reactivity is partly reduced [64]. It is acknowledged that heteroatom doping in carbon can enhance the electronic conductivity of carbon materials. Thus, N, S-doped graphene with oxygen-functionalized groups was prepared as cathode for NICs. It showed good capacitive capacity in NICs, with discharge capacities of ~52 and ~19 mAh g^−1^ at current densities of 0.2 A g^−1^ and 20 A g^−1^, respectively [33]. Its capacitance is derived from the pseudocapacitive reaction between the sodium ions and the oxygen-functionalized group and heteroatoms. Moreover, it was observed that heteroatom doping could improve the capacitance of graphite as cathode for NICs through extra intercalation/deintercalation reactions [66]. In situ Raman spectroscopy was conducted on a B-doped graphite cathode in the Na-ion hybrid capacitor, showing that the D-band and G-band gradually became weak during charging (Figure 6e). These changes to the G and D bands in Raman spectra suggest that anions were intercalated into B-doped graphite during the charging process. Furthermore, density functional theory (DFT) was used to investigate PF_6_ anion storage in the cathode of hybrid capacitors. The results showed that the energy barrier for PF_6_ diffusion in B-doped graphite is much lower than that for natural graphite (Figure 6f,g), which contributed to the fast anion diffusion in B-doped graphite in NICs [66]. Thus, there are two possible anion storage mechanisms for the cathode in hybrid capacitors, that is, surface-controlled pseudocapacitive redox reactions and diffusion-controlled intercalation/deintercalation redox reactions.

## 4. 2D Carbon Nanosheets for Post-Li Hybrid Capacitors

### 4.1. 2D Carbon Nanosheets as Anode for Post-Li Hybrid Capacitors

**Figure 7:**
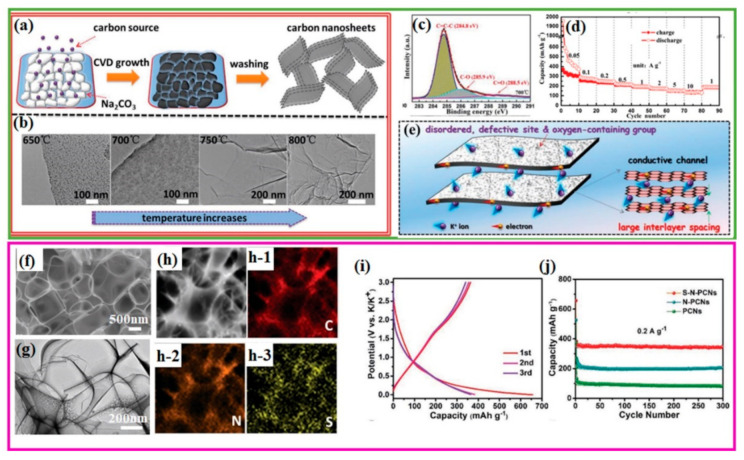
Carbon nanosheets as anode for post-Li capacitors. (**a**–**e**) Oxygen-rich carbon nanosheets (CNS) for potassium-ion hybrid capacitor: (**a**) schematic illustration of the growth of CNSs and (**b**) TEM images of CNSs obtained at different deposition temperatures, (**c**) C 1s XPS analysis of CNS deposited at 700 °C, (**d**) rate capability of CNSs, (**e**) schematic illustration of the unique features of CNS as anode material in potassium-ion batteries. Reproduced with permission [58], Copyright 2019, Wiley-VCH. (**f**–**j**) S,N-doped porous carbon nanosheets (S-N-PCNs) as anode for potassium-ion capacitors: (**f**) SEM image, (**g**) TEM image, and (**h**) EDS mapping of C, N, and S in the S-N-PCNSs, (**i**) charge–discharge curves, and (**j**) cycling performance of S-N-PCNs. Reproduced with permission [59], Copyright 2019, Wiley-VCH.

Carbon nanosheets are a key member of the 2D carbon material family, and they can be prepared from decomposition of biowastes (peanut skin [129,130] and pencil shavings [30]) and organic molecules (C_2_H_2_ gas [58], citric acid [59], ethylenediaminetetraacetic acid tetrasodium salt [107], and sodium citrate [131]). Nanostructure and dimensional design have been demonstrated as an effective approach to achieve fast sodium-ion insertion/extraction of carbon materials. It was reported that 3D architectures composed of carbon sheets are promising anode materials, which have both large surface area and short diffusion paths, thus exhibiting fast kinetics [129,130,131]. Combined with heteroatom doping and oxygen functionalization, the carbon nanosheet architecture presents excellent diffusion kinetics in hybrid capacitors [58,59,107]. Representative examples of 3D architectures composed of carbon nanosheets are summarized in Table 2. Oxygen-rich carbon nanosheets (CNSs) were prepared by chemical vapour deposition using decomposing C_2_H_2_ gas on Na_2_CO_3_ templates, as illustrated in Figure 7a. The heating temperature deeply affected the carbon nanosheet morphology, and the carbon nanosheets obtained at 800 °C showed typically graphene-like features (Figure 7b). C 1s XPS spectra showed that there were abundant oxygen groups in CNSs (Figure 7c). Owing to their unique structure and abundant exposed surfaces with disordered and oxygen-rich sites, they exhibited excellent rate capability for potassium-ion storage (Figure 7d,e). Coupled with activated carbon (AC) as cathode, a CNSs/AC potassium-ion hybrid capacitor delivered high energy density and power density of 149 Wh kg^−1^ and 21,000 W kg^−1^, respectively [58]. X. Hu et al. confirmed that the sulphur and nitrogen-doped into carbon nanosheets (S-N-PCNs) can enlarge the interlayer spaces and provide ample structural defects and redox active sites, thus improving the pseudocapacitive activity from 100 to 380 mAh g^−1^ (Figure 7j) and enabling fast kinetics towards efficient potassium-ion storage. When S-N-PCNs was used as anode and activated carbon as cathode, the AC/S-N-PCNs potassium-ion hybrid capacitor showed a high energy density of 187 Wh kg^−1^ and a high power density of 5136 W kg^−1^ [59]. Therefore, the combination of nanostructured design of carbon nanosheets, heteroatom doping, and oxygen functionalization is an effective strategy to obtain carbon anodes with excellent energy density and power density for hybrid capacitors.

### 4.2. 2D Carbon Nanosheets as Cathode for Post-Li Hybrid Capacitors

Carbon nanosheets derived from biowaste have abundant oxygen-containing functionalities and defects, thus providing extra reversible adsorption interaction of anions at surface defects and functional groups [30,129,130]. The outer skin of a peanut shell is rich in cellulosic fibrils and highly heterogeneous. These microfibrils are interlinked by a minority phase of much shorter branched polysaccharide tethers and polyphenolic polymers. Thus, the outer skin of a peanut shell is an ideal precursor to prepare carbon nanosheets with abundant surface functional groups (PSNC) through hydrothermal and chemical activation processes [130]. The XPS spectra show that there are three different types of oxygen-containing functional groups on the surface of PSNC: C=O quinone groups, C-OH phenol/C-O-C ether groups, and COOH carboxylic groups. When the PSNC was used as cathode in Na half-cells, it showed high capacitance of 119 F g^−1^, greater than commercial activated carbon with 36 F g^−1^. The excellent capacitive performance of PSNC is possibly attributable to its large surface area and mesopore content, as well as its rich oxygen content. Until now, the designed carbon nanosheet nanostructures have been applied mainly as anodes for NICs and KICs, and as cathodes for NICs, although reports are still few (Table 2). Moreover, there are no reports about the application of carbon nanosheets in zinc-ion hybrid capacitors. More investigations on the application of carbon nanosheets and other nanostructures in hybrid capacitors are needed in the future.

## 5. Graphene Composites for Post-Li Hybrid Capacitors

### 5.1. Graphene Composites as Anode for Post-Li Hybrid Capacitors

**Figure 8:**
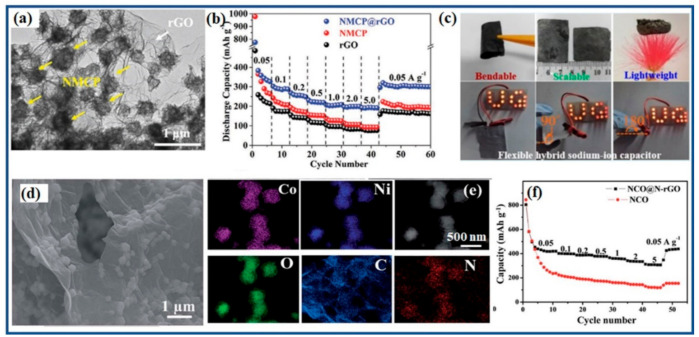
Graphene composites as anodes for post-Li hybrid capacitors. (**a**,**b**) NMCP@rGO anode for potassium-ion hybrid capacitors: (**a**) TEM image, (**b**) rate capability of NMCP@rGO potassium half-cell. Reproduced with permission [35], Copyright 2020, Wiley-VCH; (**c**) flexible sodium-ion hybrid capacitor with a graphene composite anode. Reproduced with permission [101], Copyright 2018, American Chemical Society; (**d**–**f**) NiCo_2_O_4_@N-doped rGO (NCO@N-rGO) as anode in a sodium-ion hybrid capacitor: (**d**) TEM image, (**e**) EDS mapping, and (**f**) rate performance of NiCo_2_O_4_ (NCO) and NiCo_2_O_4_@rGO. Reproduced with permission [62], Copyright 2018, Royal Society of Chemistry.

As electrode material, the tendency towards aggregation restricts the surface area of graphene, limiting its capacitance. To separate 2D graphene sheets, carbonaceous pillars (carbon nanotubes, polymers, and carbon polyhedral) are introduced into the graphene composites, which not only inhibits the graphene aggregation, but also contributes to the capacitance [32,132,133,134,135]. Ruan et al. reported a “dual-carbon” structure consisting of graphene and microporous carbon polyhedral (NMCP) derived from metal–organic frameworks (denoted as NMCP@rGO, where rGO is reduced graphene oxide) (Figure 8a), which benefited from the synergistic effect of dual-carbon and showed superior performance in K-ion hybrid capacitors. Owing to the NMCP pillars, NMCP@rGO had the largest interlayer distance of 0.378 nm compared with 0.341 nm for rGO and 0.355 nm for NMCP. As shown in Figure 8b, NMCP@rGO showed a better K-ion storage capacity of 386 mAh g^−1^ at 0.05 A g^−1^ in comparison with pure graphene or NMCP [35]. When NMCP@rGO was used as the anode and activated carbon (AC) as cathode, a NMCP@rGO/AC K-ion hybrid capacitor presented a high energy/power density (63.6 Wh kg^−1^ at 19,091 W kg^−1^). Owing to the unique 2D morphology, the graphene composite is beneficial for preparing free-standing electrodes. Zhao’s group fabricated a sodium-ion hybrid capacitor with a free-standing polyimide–graphene composite as the negative electrode and reduced graphene oxide as the positive electrode, showing a high energy density of 21.5 Wh kg^−1^ at a power density of 3400 W kg^−1^ (Figure 8c) [101].

The intercalated anodes (such as TiO_2_ [61], Nb_2_O_5_ [60], NaTi_2_(PO_4_)_3_ [65], etc.) possess poor intrinsic electronic conductivity, thus suffering primarily from a kinetic imbalance between the two electrodes that causes the entire capacitor system to collapse, with consequent poor and inferior performance. The adsorption kinetics of the carbon-based electrodes must be equivalent to that of the intercalation electrode by the storage of equivalent amounts of anions on the surface of the carbon-based electrode [68]. Graphene is used as a conductive matrix to greatly enhance the kinetics with superior sodium insertion/extraction at high current rates [65,136]. NiCo_2_O_4_ was tested as the anode electrode for sodium-ion hybrid capacitors, but it showed inferior rate performance. When encapsulated into N-doped graphene, the electronic conductivity of NiCo_2_O_4_@N-doped rGO was significantly improved due to close contact between the NiCo_2_O_4_ particles and the graphene sheets (Figure 8d,e), thus enhancing the diffusion kinetics for sodium-ion storage (Figure 8f). Using activated carbon as the cathode, the AC//NiCo_2_O_4_@N-doped rGO sodium-ion hybrid capacitor exhibited a high power density of 9750 W kg^−1^ with an energy density of 48.8 Wh kg^−1^ [62]. Examples of graphene as conductive matrix in hybrid capacitors are summarized and listed in Table 3.

### 5.2. Graphene Composites as Cathode for Post-Li Hybrid Capacitors

**Figure 9:**
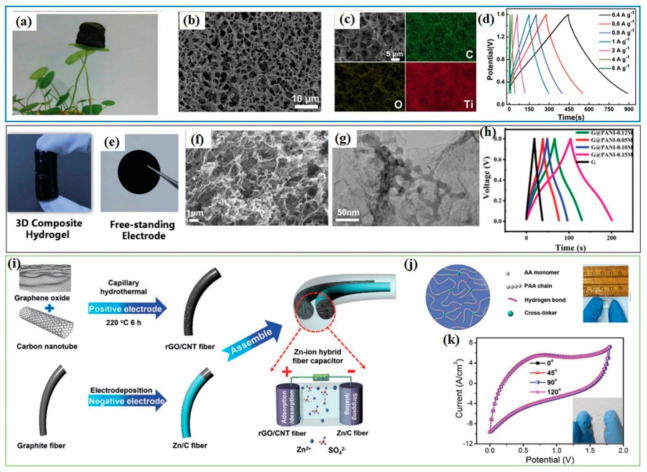
Graphene composites as cathodes for Zn-ion hybrid capacitors. (**a**–**d**) MXene@rGO aerogel for Zn-ion hybrid capacitors: (**a**) MXene-rGO aerogel standing on a piece of leaf, (**b**) SEM image, (**c**) EDS mapping, and (**d**) charge–discharge curves at current densities from 0.4 to 6 A g^−1^. Reproduced with permission [83], Copyright 2019, Wiley-VCH. (**e**–**h**) PANI@graphene hydrogel for a Zn-ion hybrid capacitor: (**e**) optical images of a 3D graphene hydrogel cylinder and the free-standing electrode film, (**f**) SEM and (**g**) TEM images of PANI@graphene, (**h**) galvanostatic charge–discharge profiles. Reproduced with permission [26], Copyright 2018, Royal Society of Chemistry. (**i**–**k**) CNT@rGO composite for Zn-ion hybrid fiber capacitors: (**i**) schematic illustration of the synthesis of CNT@rGO and the assembly of a Zn-ion hybrid fibre capacitor, (**j**) schematic illustration of a PAA hydrogel electrolyte and a photograph of a polyacrylic acid (PAA) electrolyte; (**k**) cyclic voltammetry (CV) curves of the flexible Zn-ion hybrid fibre capacitor when bent at different angles. Reproduced with permission [84], Copyright 2019, Wiley-VCH.

To date, graphene composites reported as cathodes for post-Li hybrid capacitors have mainly been applied in Zn-ion hybrid capacitors. Moreover, these reported graphene composites are prepared as flexible electrodes for flexible Zn-ion hybrid capacitors [26,83,84]. It was demonstrated that a graphene composite hydrogel with a 3D porous nanostructure can significantly enlarge the active interface area between the electrode and the electrolyte, boosting the capacity of hybrid capacitors [26,137]. MXene (Ti_3_C_2_T*_x_*)/reduced graphene oxide aerogel (MXene-rGO) was prepared by immersing rGO aerogel in an MXene nanosheet dispersion and freeze-drying, which showed the lightweight characteristic (Figure 9a). MXene-rGO presented a porous fishing net structure with the MXene uniformly attached to the rGO, as shown in Figure 9b,c. MXene-rGO aerogel was directly used as flexible cathode electrode, coupled with Zn foil to assemble Zn-ion hybrid capacitors. The charge–-discharge curves formed symmetrical triangles at various current densities, suggesting good reversibility of the MXene-rGO/Zn hybrid capacitor [83]. 3D graphene@polyaniline (PANI) hydrogel was reported as flexible cathode for Zn-ion hybrid capacitors, exhibiting excellent capacitive performance (Figure 9e–h). The PANI@rGO featured a 3D interconnected porous network with PANI particles tightly anchored on the graphene (Figure 9f,g). This unique structure combined the properties of good interface effects (e.g., large surface area, short diffusion path) and the good conductivity of graphene, thus promoting the transport of charges and ions, so that it demonstrated better capacitive performance than bare graphene aerogel (Figure 9h). Besides flexible capacitors, graphene composites can be easily used to assemble fibre-shaped capacitors, which can be manufactured to be suitable for integrating into weavable textiles. Zhang et al. reported a fiber-shaped Zn-ion hybrid capacitor with rGO/CNT composite fiber as cathode [84]. rGO/CNT fiber was prepared by hydrothermally assembling in capillary columns using CNT and graphene oxide, as illustrated in Figure 9i. It is the limitation of the liquid electrolyte that challenges the utilization of Zn-ion capacitors in flexible and wearable devices. Hence, it is imperative to present new design chemistries for Zn-ion capacitors using solid/quasi-solid electrolytes to show their possible application in wearable electronics. Neutral ZnSO_4_-filled polyacrylic acid hydrogel was shown to act as such a quasi-solid-state electrolyte, which offered high ionic conductivity and excellent stretchability (Figure 9j). The assembled fibre-shaped Zn-ion hybrid capacitor delivers a high energy density of 48.5 mWh cm^−3^ at a power density of 179.9 mW cm^−^^3^, with excellent mechanical flexibility under different deformations (Figure 9k). Due to their low cost and direct utilization of Zn metal as anode, Zn-ion hybrid capacitors are possibly promising and attractive energy storage devices. At present, the corresponding research is still in its infancy, and more investigations on developing new cathode materials need to be undertaken.

## 6. Current Issues for 2D Carbon Materials for Post-Li Hybrid Capacitors

### 6.1. Issue of the Massive Production of 2D Carbon Materials

The massive production of 2D carbon materials is the prerequisite for their wide application. “Massive scale” in industry is typically a kilogram of powder or suspension containing graphene flakes that can be produced in one batch, which has controllable and uniform properties. On the contrary, scotch tape and Hummer’s method are only suitable for lab scale as only milligrams of sample could be obtained each time. The following factors must be considered for the massive production of 2D carbon materials: (i) how to achieve the desired properties and morphology for target graphene products; (ii) the scalability (or feasibility) from laboratory to industry; and (iii) the overall cost and environmental requirements. According to a review released by Cheng et al., there were three commercialized routes towards producing 2D carbon materials: direct liquid-phase exfoliation of graphite; oxidization of graphite and the subsequent exfoliation and/or reduction; and chemical vapour deposition (CVD) [138]. However, each method has its own problems: direct and subsequent exfoliation may present the problem of waste liquid, while the CVD route requires high facility input and precise parameter control. Therefore, there is still an urgent need for new, low-cost, and greener synthesis routes for 2D carbon materials.

### 6.2. Issue of the Stability of Carbon Materials

The stability of carbon-based materials in a battery system has long been a big issue, especially in the Na/Li–O_2_ system, in which the high operation voltage and presence of superoxide ions will corrode the carbon electrode [139]. However, the stability of carbon in a supercapacitor has been rarely mentioned as most of the researchers believe that carbon materials are super-stable in supercapacitors, as proved by the excellent cycle performance shown in Table 1, Table 2 and Table 3. Unfortunately, the stability of carbon materials in a supercapacitor also depends on conditions. If used as electrode only in the low potential (<2 V) range, the carbon materials will be very stable [140,141]. By contrast, for some high-voltage supercapacitors (>4 V), which use ionic liquids (ILs) as electrolyte, it is still possible that carbon will react with certain ILs due to high potential [142]. If used as an anion adsorption/desorption cathode, carbon materials will be stable. However, once 2D carbon materials are used as an insertion-type anode, repeated insertion/desertion of cations during charge/discharge may lead to the collapse and aggregation of the 2D structure, leading to deteriorated performance of the supercapacitor, even in low-potential capacitors [51]. In most cases, the thermal and electrical conductivity of carbon materials are excellent; the carbon materials should be stable. However, although not common, heat may be produced and accumulated if these properties are not so “good”, especially on discharge/charge with an extremely high current density. The heat and high voltage may promote the corrosion of carbon if a strong alkaline (such as 6 M KOH) electrolyte is used ($C-4e^{-}+4OH^{-}\to CO_{3}^{2-}+H_{2}O+2H^{+}$). Hence, the stability of carbon, although could be ignored in most cases, may be an issue under some conditions.

### 6.3. Issue of the Kinetic Balance between the Two Electrodes

Rather than the mass balance between the electrodes, it is the kinetic imbalance between the two electrodes that causes the entire capacitor system to collapse, with consequently poor and inferior performance. Therefore, it is important to balance the kinetics of the cathode and the anode to fabricate a stable and efficient hybrid capacitor system [143].

The intercalation kinetics of the intercalated electrode must be equivalent to that of the adsorption electrode by storage of equivalent amounts of cations in the intercalation-type electrode [68]. Generally, the intercalated anodes have poor intrinsic electronic conductivity. Therefore, one approach to overcome the kinetic limitation of intercalated-type electrodes is by increasing the electrical conductivity of these electrodes via introducing highly conductive additives. Graphene has a high surface area (>2600 m^2^ g^−1^), unique physicochemical properties, and ultrahigh electronic conductivity [65]. Graphene is usually used as a conductive matrix to form graphene composites, thus greatly enhancing the kinetics of cation insertion/extraction at high current rates [65,136]. Secondly, to increase the kinetics of carbon materials, nanostructure design is an effective approach, such as 3D porous structures and fine constructed nanoarrays. In addition, the functionalization and heteroatom doping on a carbon lattice could also enhance the kinetics of carbon materials.

### 6.4. Issues for the Electrolyte

The electrolyte, as an important component of hybrid capacitors, plays a crucial role by providing ionic charge carriers for the charge and discharge process. To improve the electrochemical performance of hybrid capacitors, the electrolyte must have high ion conductivity, low viscosity, good thermal stability, and a wide potential window. At present, the electrolytes explored for hybrid capacitors include organic electrolyte, aqueous electrolyte, and quasi-solid-state electrolyte [144]. There are some issues related to the electrolyte. In the organic electrolyte system, porous structures with large specific surface areas tend to incur severe electrolyte decomposition to form a thick solid electrolyte interphase (SEI), resulting in low initial Coulombic efficiency. Moreover, sometimes the SEI film that is formed is unstable, causing continuous electrolyte decomposition and low Coulombic efficiency. To form a suitable and stable SEI layer, usually fluoroethylene carbonate (FEC) is used as an electrolyte additive. Although organic electrolytes have been widely used, their volatility and flammability remarkably limit their practical applications in hybrid capacitors. Thus, developing new electrolytes, including aqueous and quasi-solid-state electrolytes, is urgent.

Zn-ion hybrid capacitors, which commonly use aqueous electrolytes, suffer from the issues of a narrow electrochemical window (caused by the decomposition of water at ~1.23 V) and low Zn stripping/plating efficiency. The electrolyte is a key component of Zn-ion hybrid supercapacitors, and its capability of enabling high Zn stripping/plating efficiency is essential to the cycling stability of the metallic Zn anode and high-performance Zn-based hybrid supercapacitors [31]. The concept of “water-in-salt” (WIS) has been applied to expand the working window of the aqueous electrolyte. Wang’s group reported that lithium-ion water-in-salt electrolyte (LiTFSI) solutions at a concentration of 21 M exhibited superior electrochemical properties over a wide working voltage window of 3 V [145,146]. Moreover, the highly concentrated electrolyte was found to improve the initial Coulombic efficiency of the Zn-ion hybrid capacitor [31]. Thus, the “water-in-salt” electrolyte could widen the electrochemical window and improve the Zn stripping/plating efficiency. The “water-in-salt” electrolyte, however, increases the production cost, due to the utilization of a high salt concentration. Therefore, more investigations are required to develop new electrolytes with a wide voltage window, high ionic conductivity, and high safety.

## 7. Summary and Outlook

In this conceptual review, we have summarized the recent progress on the application of 2D carbon materials in post-Li hybrid capacitors, including sodium-ion hybrid capacitors, potassium-ion hybrid capacitors, and zinc-ion hybrid capacitors. This article provides a comprehensive overview of the mechanisms and variety of hybrid capacitors, the functions of 2D carbon materials in hybrid capacitors, and current problematic issues for hybrid capacitors. The storage mechanism of hybrid capacitors is based on both non-faradaic cathodes (based on anion adsorption on their surfaces) and faradaic anodes (based on redox reactions of the cations). According to the valence of cation charge carriers, the hybrid capacitors can be divided into two types: one comprises the alkali-metal-ion hybrid capacitors, including Li-ion hybrid capacitors, Na-ion hybrid capacitors and K-ion hybrid capacitors; the other comprises the multivalent metal-ion hybrid capacitors, including Zn-ion hybrid capacitors, Mg-ion hybrid capacitors, Ca-ion hybrid capacitors and Al-ion hybrid capacitors. In hybrid capacitors, 2D carbon materials can play several roles such as cathode electrodes, anode electrodes and conductive matrices to greatly enhance the kinetics of electrodes.

Currently, the application of 2D carbon materials in post-Li hybrid capacitors suffers from the following issues: (1) The difficulty in massive production and stability of carbon materials; (2) the kinetic imbalance between the two electrodes causing the entire capacitor system to collapse, with consequently inferior performance; (3) some issues related to the electrolyte resulting in low initial Coulombic efficiency and a narrow electrochemical window. To overcome the kinetic limitations of 2D carbon electrodes, there are some strategies including: (i) increasing the electrical conductivity of the electrodes by introducing highly conductive additives; (ii) nanostructure design, such as 3D porous structures and fine constructed nanoarrays; (iii) functionalization and heteroatom doping on the carbon lattice. To overcome the issues for the electrolyte, some approaches that have been reported can be summarized into two aspects: (i) introducing electrolyte additives (such as fluoroethylene carbonate (FEC)) to form a stable SEI layer; and (ii) increasing the salt concentration to widen the voltage window of an aqueous electrolyte.

Post-Li hybrid capacitors are expected to play important roles in energy storage fields that can benefit from their high energy densities and high power densities. 2D carbon materials (graphite, graphene, carbon nanosheets) are promising candidates for hybrid capacitors, owing to their unique physical and chemical properties, including their enormous specific surface area, abundant active sites (surface and functional groups) and large interlayer spacing. Nevertheless, the utilization of 2D carbon materials in post-Li hybrid capacitors is still in its early stages, and further research and exploration are necessary. In the future, more investigations are required to develop new electrolytes with wide voltage windows, high ionic conductivity, and high safety. Moreover, it is necessary to develop new capacitor-type electrodes that can work without a binder or a conductive additive, which increase the total weight and thus decrease the final specific capacitance of the devices.

## Figures and Tables

**Table 1:** Graphite/graphene for post-Li hybrid capacitors.

Materials	Function	Counter-Electrode	Electrolyte	Energy Density (Wh kg^−1^)	Power Density (W kg^−1^)	Reported Cycles	Capacitance
Natural graphite [45]	Anode for NIC	Activated carbon	NaPF_6_/Diglyme	21.8	17,127	5000 at 15 A/g	83 mAh/g at 2 A/g
B-doped graphite [66]	Cathode for NIC	Hollow carbon	NaPF_6_/EC-DMC	108	495	2000 at 1 A/g	114 mAh/cm^3^ at 0.05 A/g
Graphene [65]	Cathode for NIC	NaTi_2_(PO_4_)_3_/graphene	NaClO_4_ in organic solvent	---	---	75,000 at 4 A/g	200 mAh/g at 0.1 A/g
Oxygen-functionalized graphene [44]	Anode/cathode for NIC	Same	Gel polymer electrolyte	121.3	300	2500 at 1 A/g	460 mAh/g at 20 mA/g
N,S-doped graphene hollow spheres [33]	Anode/cathode for NIC	Same	NaClO_4_/EC-DEC	69	51,000	10,000 at 5 A/g	272 mAh/g at 0.5 A/g
Natural graphite [45]	Anode for KIC	Activated carbon	KPF_6_/Diglyme	18.8	15,887	5000 at 15 A/g	80 mAh/g at 2 A/g
N,P-doped graphene [46]	Anode for KIC	Activated carbon	KPF_6_/EC-DEC	41.6	14,976	500 at 0.5 A/g	859 mAh/g at 0.1 A/g

**Table 2:** Carbon nanosheets for post-Li hybrid capacitors.

Materials	Function	Counter-Electrode	Electrolyte	Energy Density (Wh kg^−1^)	Power Density (W kg^−1^)	Reported Cycles	Capacitance
Peanut skin derived carbon nanosheet [129]	Cathode/anode for NIC	Same	NaClO_4_/EC-DEC	45	12,000	3000 at 5 A/g	461 mAh/g at 0.1 A/g
Peanut shell carbon nanosheet [130]	Cathode/anode for NIC	Same	NaClO_4_/EC-DEC	50	16,500	100,000 at 51.2 A/g	161 mAh/g at 0.1 A/g
3D architectures composed of carbon sheets [131]	Anode for NIC	Activated 3D architecture composed of carbon sheets	NaClO_4_/DMC	111	200	10,000 at 10 A/g	400 mAh/g at 0.1 A/g
Oxygen-rich carbon nanosheets [58]	Anode for KIC	Activated carbon	KPF_6_/EC-DEC	149	21,000	3000 at 5 A/g	369 mAh/g at 0.05 A/g
3D architectures composed of N-doped carbon nanosheets [107]	Anode for KIC	Activated 3D architecture composed of N-doped carbon nanosheets	KPF_6_/EC-DMC	76.4	21,000	10,000 at 2 A/g	207 F/g at 1 A/g
S,N-doped 3D porous carbon nanosheet [59]	Anode for KIC	Activated carbon	KPF_6_/EC-DEC	187	5136	6000 at 2 A/g	107 mAh/g at 20 A/g

**Table 3:** Graphene composites for post-Li hybrid capacitors.

Materials	Function	Counter-Electrode	Electrolyte	Energy Density (Wh kg^−1^)	Power Density (W kg^−1^)	Reported Cycles	Capacitance
Polyimide/graphene composite [101]	Anode for NIC	Graphene	NaClO_4_/EC-PC-0.3%FEC	55.5	395	200 at 25 mA/g	225 mAh/g at 100 mA/g
NaTi_2_(PO_4_)_3_/graphene [65]	Anode for NIC	Graphene	NaClO_4_	80	8000	75,000 at 4 A/g	118 F/g at 0.15 A/g
Nb_2_O_5_/graphene [60]	Anode for NIC	Activated carbon	NaPF_6_/EC-DMC+5%FEC	76	20,800	3000 at 1 A/g	750 mAh/g at 0.025 A/g
MoS_2_/rGO [87]	Anode for NIC	N-doped 3D graphene	NaClO_4_/EC-DMC	43	103,000	10,000, no rate	585 mAh/g at 0.1 A/g for Na
TiO_2_/graphene [61]	Anode for NIC	Activated carbon	NaClO_4_/EC-PC+5%FEC	25.8	1367	10,000 at 3.35 A/g	162 mAh/g at 1.675 A/g
NiCo_2_O_4_/N-doped rGO [62]	Anode for NIC	Activated carbon	NaPF_6_/diethylene glycol dimethyl ether	48.8	9750	100 at 0.1 A/g	439 mAh/g at 0.05 A/g
Nitrogen-doped carbon polyhedron@ rGO [35]	Anode for KIC	Activated carbon	KPF_6_/EC-DEC	63.6	19,091	6000 at 5 A/g	351 mAh/g at 0.05 A/g
Nitrogen-doped MoSe_2_/graphene [63]	Anode for KIC	Activated carbon	KPF_6_/EC-DEC	119	7212	3000 at 1 A/g	401 mAh/g at 0.2 A/g
Co_2_P nanorod/graphene [108]	Anode for KIC	Activated carbon	KPF_6_/EC-DEC-EMC	87	4260	5000 at 0.2 A/g	374 mAh/g at 20 mA/g
MXene-rGO aerogel for ZIB hybrid [83]	Cathode for ZIC	Zn foil	2M ZnSO_4_	34.9	279.9	75,000, no rate	128.6 F/g at 0.4 A/g
rGO/CNT for flexible zinc-ion hybrid capacitor [84]	Cathode for ZIC	Zn foil	ZnSO_4_/PAA hydrogel	48.5 mWh/cm^3^	179.9 mW/cm^3^	10,000 at 3.2 A/cm^3^	104.5 F/cm^3^ at 0.4 A/cm^3^
Binder-free PANI@graphene [26]	Cathode for ZIC	Zn foil	2M ZnSO_4_	138	2455	6000 at 0.1 A/g	154 mAh/g at 0.1 A/g
